# Perioperative Respiratory Outcome of Patients with Eosinophilia: A Cohort Study in a Tertiary Care Hospital

**DOI:** 10.1155/2023/8514949

**Published:** 2023-01-10

**Authors:** Nari M. Lyngdoh, Rajani Thabah, Sunny Aggarwal, Laltanpuii Sailo, Raju Shakya, Julie Wahlang, Badondor Shylla, Chhandasi Naskar

**Affiliations:** ^1^Department of Anaesthesiology and Critical Care, NEIGRIHMS, Mawdiangdiang, Shillong 793018, Meghalaya, India; ^2^Department of Pharmacology, Mawdiangdiang, Shillong 793018, Meghalaya, India; ^3^Nursing College, NEIGRIHMS, Mawdiangdiang, Shillong 793018, Meghalaya, India

## Abstract

**Background:**

A respiratory adverse event is one of the main causes of critical events in the perioperative period. Perioperative distress symptoms like cough and stridor have been reported to occur in patients with hyperreactive airways.

**Objective:**

This study was conducted to determine the relationship between blood eosinophil count and perioperative adverse respiratory events among different age groups of patients who require general anesthesia for different types of surgeries.

**Methods:**

A cohort study was conducted on 197 patients of either gender, aged 3 years and above, belonging to ASA classes I–II, who were scheduled to undergo surgery requiring general anesthesia and intubation. Patients were stratified according to absolute eosinophil count into two groups: Group A (AEC 0 to 499/mm^3^) and Group B (AEC 500 to 1000/mm^3^). Patients were monitored for 24 hours in the perioperative period for adverse respiratory events such as bronchospasm, laryngospasm, a fall in SPO2 < 95%, and cough and stridor.

**Results:**

A total of 197 patients were evaluated, with a median age of 37 ± 14.4 years. The percentage range of adverse respiratory events across different age groups was 35% in adults to 60% in children. Major complications noted were a fall in SPO2 < 95% (62.5%) and cough (27.7%) as per CTCAE v5.0 (November 27, 2017). The Naranjo score of adverse respiratory events was categorized as possible with mild level 1 severity. Adverse respiratory events were managed with humidified oxygen, antitussives, and bronchodilators.

**Conclusions:**

Eosinophilia is seen in one-third of the patients undergoing surgical interventions. Patients with a blood eosinophil count of ≥400/mm^3^ had an increased risk of exacerbations of respiratory adverse events in the perioperative period.

## 1. Introduction

In patients receiving general anesthesia, respiratory adverse events are one of the main causes of critical events occurring in the perioperative period. Perioperative distress symptoms like cough and stridor have been reported to occur in patients with hyperreactive airways [1, 2]. The absolute eosinophil count (AEC) in the peripheral blood is in the range of 350 and 500/mm^3^, with a corresponding range of 3–5% of the total white blood cell count. Eosinophilia is considered when there is an increase in AEC from 500/mm^3^ [3, 4]. Eosinophils can release mediators such as cytokines, leukotrienes, and chemokines, which lead to inflammation, and eosinophil granule-specific cationic proteins, which play a role in host defense mechanisms against helminths, viruses, and bacterial organisms [5, 6].

These mediators and cationic proteins have also been implicated in inducing mucosal damage and the development of bronchial hyperresponsiveness that clinically manifests as respiratory spasm and oxygen desaturation [7]. Obstructive pulmonary disorders are associated with different prognoses, especially during the perioperative period. One of the most widely studied phenotypes of such disorders is an increased eosinophil count. The role of the blood eosinophil count as a marker of risk of exacerbation of adverse perioperative complications has been reported as a beneficial tool to identify high-risk patients prone to developing adverse respiratory symptoms. Patients with an eosinophil count ≥400 cells/*μ*L were predicted to have at least two episodes of airway distress in a year, and the correlation between sputum eosinophil levels and airway hyperreactivity has been demonstrated by several studies [8–12]. Therefore, this study aims to assess the relationship between blood eosinophil count and adverse perioperative respiratory events among different age groups of patients who require general anesthesia for different types of surgeries.

## 2. Materials and Methods

This was a case-cohort study conducted in a tertiary care center to determine the relationship between an absolute eosinophil count (AEC) and adverse perioperative respiratory events among different age groups. The study was approved by the institution's Ethics Committee (IEC, NEIGR/IEC/M3/F4/17).

  Sample size: the minimum calculated sample size as calculated by OpenEpi version 3 is 147 with 5% precision and 95% confidence level (prevalence of 10.7%[13]).
  Study design: the study was conducted on patients who were scheduled to undergo elective surgery requiring general anesthesia and intubation. After obtaining informed consent from the patients of either gender, those belonging to the American Society of Anesthesiology (ASA) physical status I and II were included in the study. Patients with cardiac disease, a history of respiratory tract infection within two weeks prior to surgery, a difficult airway requiring more than three intubation attempts, airway surgeries, or an AEC greater than 1000 mm^3^ were excluded from the study.

All patients underwent a routine preanesthetic check-up, which included taking a medical history, surgical history, allergy history, current treatment, a detailed physical examination, laboratory and radiological investigations, American Society of Anesthesiology (ASA) grading, and an anesthesia plan. Routine blood work was also carried out, which included a total and differential leukocyte count.

The calculation of the absolute eosinophil count is as follows:

(1) $$\begin{array}{c} AEC=percentage\;of\;eosinophil\times total\;leukocyte\;count\text{.} \end{array}$$

Stratification of patients according to their absolute eosinophil count: I **Group A**: AEC = 0 to 499/mm^3^ and II **Group B**: AEC = 500 to 1000/mm^3^

### 2.1. Perioperative and Postoperative Monitoring

Heart rate, electrocardiogram, noninvasive blood pressure, pulse oximetry, and capnography were monitored. A standard anesthesia technique was applied. Intravenous injections of propofol (2 mg/kg IV), fentanyl (2 *µ*g/kg IV) and vecuronium 0.1 mg/kg IV were used for induction of anesthesia. The patient was maintained with oxygen and isoflurane. At the end of the surgery, reversal was carried out using an injection of neostigmine (0.5 mg/kg IV) and an injection of Glycopyrrolate (0.008 mg/kg IV).

In the postoperative period, patients were monitored in the recovery room for 2 hours and thereafter in the ward for 1, 2, 12, and 24 hours. Any adverse respiratory event that occurred in the perioperative period was noted. This includes any or all of the following as per CTCAE v5.0 (November 27, 2017).

1. Bronchospasm: characterized by a sudden contraction of the smooth muscles of the bronchial wall.
2. Laryngospasm: characterized by paroxysmal spasmodic muscular contraction of the vocal cords.
3. Cough: a sudden, often repetitive, spasmodic contraction of the thoracic cavity resulting in the violent release of air from the lungs and usually accompanied by a distinctive sound.
4. Stridor: characterized by a high-pitched breathing sound due to laryngeal or upper airway obstruction.
5. A fall in SPO2 of less than 95%.

## 3. Results

A total of 197 patients who fulfilled the inclusion criteria were enrolled in the study. The mean age was 37 ± 14.4 years, out of which female patients accounted for 69.2% and 30.5% were males (Table 1). The most common surgery for which anesthesia was given was open and laparoscopic cholecystectomy (79%). There was a total of 72 (36.5%) adverse respiratory events viz. fall in SPO2 level <95% (62.5%) and cough (27.7%) of the total adverse events, respectively. Most of the adverse events were noted with an AEC of less than 500/mm^3^. Patients in the age group of fewer than 18 years with an AEC of 500/mm^3^ had more respiratory complications than adults (Tables 2 and 3). Causality and severity assessment score of less than 3 (Possible) with mild level 1 severity as analysed by the Naranjo probability scale and Modified Hartwig and Siegel Severity scale (Table 4) [14, 15].

## 4. Discussion

The main aim of this study was to determine the relationship between eosinophil count and adverse perioperative respiratory events among different age groups.

In our study, the incidence of eosinophilia was 16.24% with an AEC of >500/mm^3^ (*n* = 32). A retrospective study conducted by Schleich et al. among 508 asthmatics reported that 211 patients had eosinophilic counts (≥3% eosinophils), and they also showed the presence of IgE-mediated hypersensitivity events. They concluded that blood eosinophils can be used to predict sputum eosinophilia [8]. Louis et al. reported the association of mild to moderate asthma patients to with eosinophilia; however its role in airway hyperresponsiveness is lacking [16]. A study reported by Bansal et al. showed that among hospital attendees, 10.7% of patients had eosinophilia ranging from mild (62%) to severe (0.3%); the majority of them were males (71.2%), with ages ranging from 3 to 74 years [13].

In our study, we evaluated the blood eosinophil count, and around 2% of our study population had a history of allergies. Many other studies have found that increased levels of eosinophils are associated with bronchial asthma, allergic reactions, parasitic infections, neoplasms, collagen vascular disorders, and hypereosinophilic syndrome [17]. Eosinophilia, which is associated with helminthic infections, can be treated with antihelminthic drugs [18].

The present study showed a higher frequency of eosinophilia in adults as compared to children, which is similar to a study conducted by Bansal et al. [13]. This is in contrast to a study reported by Sreedharanunni et al. that found patients under the age of 18 years constituting one-third of the study population [19]. Madhumitha et al., in their study population of 1341 hospitalized pediatric patients, found that 12.6% had eosinophilia, with 82% having mild eosinophilia and 17% having a moderate type of eosinophilia commonly found in the age group of 10–12 years [20]. The major limitation in our study is that our study population is limited to surgical patients, and as such, the incidence of pediatric population was lower.

In our study, we noted that the respiratory complication most commonly encountered was a fall in oxygen saturation (27.4%) and cough (10.2%) within the first 12 hours of the postoperative period. In a similar report by Warner et al., out of 1,547 cases that were diagnosed with definite asthma and had undergone at least one surgical procedure, 12 patients developed bronchospasm, and two patients had laryngospasm, which were managed by bronchodilators and recovered [1]. Gurrieri et al. reported anaphylactic reactions among 38 patients during the perioperative period, which occurred during the different stages of anesthesia, including the recovery stage, and ranged from 73.7% to 7.9% with respiratory events like bronchospasm and hypoxia [21]. In our hospital, we have seen that two patients who had eosinophilia (>500/mm^3^) developed bronchospasm intraoperatively (Grade 3, CTCAE v 5.0—November 27, 2017) and only one patient who had an eosinophil count of >400/mm^3^ developed laryngospasm after extubation (Grade 3, CTCAE v 5.0—November 27, 2017), which may suggest that vigilance regarding patients with an eosinophilia count of >400/mm^3^ is warranted. We report that all the patients were managed with humidified oxygen and bronchodilators and were subsequently discharged with no other untoward events.

Eosinophilia can also be associated with the drug rash with eosinophilia and systemic symptoms (DRESS) syndrome or hypereosinophilic syndrome (HES). DRESS syndrome is a severe idiosyncratic drug reaction that is potentially life-threatening [22]. When the absolute eosinophil count is greater than 1500/mm^3^, it is known as Hypereosinophilic syndrome (HES). HES is associated with organ involvement due to eosinophilic infiltration into tissues, which can also be life-threatening if it causes myocardial damage, pulmonary involvement with hypoxia, or neurological involvement. High-dose corticosteroid therapy is recommended [23]. In our study, the upper limit for AEC was 1000 mm^3^, so HES was not observed. DRESS syndrome was also not observed in any patient during the period of observation.

### 4.1. Limitations

The limitation of this study is that most of our patients were adults. Children comprised only a small percentage. This might account for the fact that there is no statistically significant association between AEC and respiratory complications in children, despite their suffering more adverse events than adults. We also did not include patients with eosinophil counts higher than 1000/mm^3^. As group B patients with high AEC values displayed a higher incidence of respiratory complications, it is likely that, had we included patients with an AEC greater than 1000 mm^3^, we might have found a significant association between AEC and adverse perioperative respiratory events.

## 5. Conclusion

To conclude, we find that there is no significant association between AEC and adverse perioperative respiratory events; therefore, an eosinophil count of up to 1000 mm^3^ is not a risk factor for adverse perioperative respiratory events in any age group nor any other perioperative complications. Patients with a blood eosinophil count of ≥400/mm^3^ had an increased risk of exacerbations of respiratory adverse events in the perioperative period. However, further research can be undertaken for a larger group of patients with higher AEC counts. The outcome of such studies would bring about a concrete conclusion to our findings.

## Figures and Tables

**Table 1:** Demographic, allergic, and history of substance use of the respondents (*N*  = 197).

Parameters	*N* (%)
Gender	
Male	60 (30.5)
Female	137 (69.2)
Age (year) median with interquartile range	37 ± 14.4 (median range = 27.49)
^*∗*^ASA physical status	
I	109 (55.3)
II	88 (44.7)
History of allergy	4 (2.0)
History of smoking	31 (15.7)
Alcohol use	7 (3.5)

^*∗*^ASA: American Society of Anesthesiology.

**Table 2:** Relationship between different age groups and adverse respiratory events.

Age	AEC	Respiratory complications (*N* = 72)	
		Yes	No
<5 years–18 years (*N* = 12)	Up to 499/mm^3^ (*N* = 7)	3	4
	>500 to 1000/mm^3^ (*N* = 5)	3^*∗∗*^	2^*∗∗*^

>18 years (*N* = 185)	Up to 499/mm^3^(*N* = 158)	57 (36.07)	101 (63.92)
	>500 to 1000/mm^3^ (*N* = 27)	9 (33.33)^*∗∗*^	18 (66.67)^*∗∗*^

^*∗∗*^AEC > 500/mm^3^, *N* = 32 (16.2%).

**Table 3:** Association between absolute eosinophil count and adverse respiratory events.

AEC categories	Respiratory complications (CTCAE v 5.0—November 27, 2017)				
	Fall in oxygen saturation (SPO2 <95%)	Cough	Stridor	Bronchospasm	Laryngospasm
Group A (*N* = 165) AEC 0–499/mm^3^	37	17 (Grade 1-2)	4 (Grade 3)	0	1 (Grade 3)
GP B (*N* = 32) AEC 500–1000/mm^3^	8	3 (Grade 1-2)	0	2 (Grade 3)	0
Perioperative period	12 hours postoperative period	10 hours postoperative period	12 hours postoperative period	Intraoperative period	Immediate after extubation
Outcome	Surgery was completed, and the patient was managed with humidified oxygen in the ward	Surgery was completed, and the patient was managed with antitussives	Surgery was completed, and the patient was managed with intravenous steroids and nebulised adrenaline	Surgery was completed, and the patient was managed with intravenous bronchodilators	Surgery was completed, and the patient was managed with jaw thrust and positive pressure ventilation with 100% oxygen

**Table 4:** Causality and severity assessment of adverse drug reactions using the Naranjo probability scale and the Modified Hartwig and Siegel Severity scale [10, 11].

Adverse drug events (*N* = 72)	Possible	Severity
Sore throat	20	Mild
		Level 1

Cough	2	Mild
		Level 1

Bronchospasm	4	Mild
		Level 1

Stridor	1	Mild
		Level 1

Fall in oxygen saturation (SPO2 <95%)	45	Mild
		Level 1

## Data Availability

The data used to support the findings of this study can be obtained from the corresponding author upon request.

## References

[1] Warner D. O., Warner M. A., Barnes R. D. (1996). Perioperative respiratory complications in patients with asthma. *Anesthesiology*.

[2] Parameswara G. (2015 Jan 1). Anesthetic concerns in patients with hyper-reactive airways. *Karnataka Anaesthesia Journal*.

[3] Shomali W., Gotlib J. (2019). World Health Organization‐defined eosinophilic disorders: 2019 update on diagnosis, risk stratification, and management. *American Journal of Hematology*.

[4] Leru P. M. (2019). Eosinophilic disorders: evaluation of current classification and diagnostic criteria, proposal of a practical diagnostic algorithm. *Clinical and Translational Allergy*.

[5] Travers J., Rothenberg M. E. (2015). Eosinophils in mucosal immune responses. *Mucosal Immunology*.

[6] Ravin K. A., Loy M. (2016). The eosinophil in infection. *Clinical Reviews in Allergy and Immunology*.

[7] Lee L. Y., Gu Q., Lin A. H., Khosravi M., Gleich G. (2019). Airway hypersensitivity induced by eosinophil granule-derived cationic proteins. *Pulmonary Pharmacology and Therapeutics*.

[8] Schleich F. N., Manise M., Sele J., Henket M., Seidel L., Louis R. (2013 Dec). Distribution of sputum cellular phenotype in a large asthma cohort: predicting factors for eosinophilicvsneutrophilic inflammation. *BMC Pulmonary Medicine*.

[9] Miravitlles M., Soler-Cataluña J. J., Soriano J. B. (2022). Determinants of blood eosinophil levels in the general population and patients with COPD: a population-based, epidemiological study. *Respiratory Research*.

[10] Vogelmeier C. F., Kostikas K., Fang J. (2019). Evaluation of exacerbations and blood eosinophils in UK and US COPD populations. *Respiratory Research*.

[11] Saha S., Brightling C. E. (2006). Eosinophilic airway inflammation in COPD. *International Journal of COPD*.

[12] Gotlib J., Cross N. C., Gilliland D. G. (2006). Eosinophilic disorders: molecular pathogenesis, new classification, and modern therapy. *Best Practice and Research Clinical Haematology*.

[13] Bansal R., Kaur A., Suri A. K., Kaur P., Bansal M., Kaur R. (2017). Incidence of eosinophilia in rural population in north India: a study at tertiary care hospital. *Annals of Pathology and Laboratory Medicine*.

[14] Zaki S. (2011). Adverse drug reaction and causality assessment scales. *Lung India*.

[15] Srinivasan R., Ramya G. (2011). Adverse drug reaction-causality assessment. *International Journal of Research in Pharmacy and Chemistry*.

[16] Louis R., Sele J., Henket M. (2002). Sputum eosinophil count in a large population of patients with mild to moderate steroid-naive asthma: distribution and relationship with methacholine bronchial hyperresponsiveness. *Allergy*.

[17] Stella S., Massimino M., Manzella L. (2021). Molecular pathogenesis and treatment perspectives for hypereosinophilia and hypereosinophilic syndromes. *International Journal of Molecular Sciences*.

[18] Insiripong S., Siriyakorn N. (2008). Treatment of eosinophilia with albendazole. *Southeast Asian Journal of Tropical Medicine and Public Health*.

[19] Sreedharanunni S., Varma N., Singh Sachdeva M. U. (2018). The spectrum of hypereosinophilia and associated clonal disorders–a real world data from a tropical setting. *Mediterranean Journal of Hematology and Infectious Diseases*.

[20] Madhumitha S., Eswaran Y., Jayaganesh P. (2019). Study on peripheral eosinophilia among paediatric patients in a tertiary care hospital. *Journal of Clinical and Diagnostic Research*.

[21] Gurrieri C., Weingarten T. N., Martin D. P. (2011). Allergic reactions during anesthesia at a large United States referral center. *Anesthesia and Analgesia*.

[22] Choudhary S., McLeod M., Torchia D., Romanelli P. (2013). Drug reaction with eosinophilia and systemic symptoms (DRESS) syndrome. *The Journal of clinical and aesthetic dermatology*.

[23] Roufosse F., Weller P. F. (2010). Practical approach to the patient with hypereosinophilia. *The Journal of Allergy and Clinical Immunology*.

